# Therapeutic Alliance as Active Inference: The Role of Therapeutic Touch and Biobehavioural Synchrony in Musculoskeletal Care

**DOI:** 10.3389/fnbeh.2022.897247

**Published:** 2022-06-30

**Authors:** Zoe McParlin, Francesco Cerritelli, Giacomo Rossettini, Karl J. Friston, Jorge E. Esteves

**Affiliations:** ^1^Clinical-Based Human Research Department, Foundation COME Collaboration, Pescara, Italy; ^2^School of Physiotherapy, University of Verona, Verona, Italy; ^3^Institute of Neurology, Wellcome Centre for Human Neuroimaging, London, United Kingdom; ^4^Malta ICOM Educational, Gzira, Malta; ^5^University College of Osteopathy, London, United Kingdom

**Keywords:** therapeutic alliance, active inference, touch, manual therapy, physiotherapy, osteopathy, musculoskeletal care, pain

## Abstract

Touch is recognised as crucial for survival, fostering cooperative communication, accelerating recovery, reducing hospital stays, and promoting overall wellness and the therapeutic alliance. In this hypothesis and theory paper, we present an entwined model that combines touch for alignment and active inference to explain how the brain develops “priors” necessary for the health care provider to engage with the patient effectively. We appeal to active inference to explain the empirically integrative neurophysiological and behavioural mechanisms that underwrite synchronous relationships through touch. Specifically, we offer a formal framework for understanding – and explaining – the role of therapeutic touch and hands-on care in developing a therapeutic alliance and synchrony between health care providers and their patients in musculoskeletal care. We first review the crucial importance of therapeutic touch and its clinical role in facilitating the formation of a solid therapeutic alliance and in regulating allostasis. We then consider how touch is used clinically – to promote cooperative communication, demonstrate empathy, overcome uncertainty, and infer the mental states of others – through the lens of active inference. We conclude that touch plays a crucial role in achieving successful clinical outcomes and adapting previous priors to create intertwined beliefs. The ensuing framework may help healthcare providers in the field of musculoskeletal care to use hands-on care to strengthen the therapeutic alliance, minimise prediction errors (a.k.a., free energy), and thereby promote recovery from physical and psychological impairments.

## Introduction

For over 50 years, the medical community has recognised the beneficial therapeutic effects of touch on health and wellbeing (McGlone et al., 2017). Consequently, hands-on care is recommended for a range of musculoskeletal (MSK) conditions, including low back pain (LBP), neck pain, headaches, carpal tunnel syndrome and hip and knee osteoarthritis (Deyle et al., 2000; Akalin et al., 2002; MacDonald et al., 2006; Rubinstein et al., 2011; Gross et al., 2015; Cerritelli et al., 2017). A significant body of evidence demonstrates that hands-on techniques in MSK care have a particularly important role on pain modulation (Deyle et al., 2000; Bialosky et al., 2009; Vigotsky and Bruhns, 2015; Cerritelli et al., 2017; Geri et al., 2019; Sánchez-Romero et al., 2021). Therefore, the hand plays a crucial role in delivering MSK care through the sense of touch. Touch in the clinical context forms part of a range of examination and therapeutic interventions such as manual therapy (Rondoni et al., 2017). It is also central to enhancing communication, patient compliance, cueing and assisting patients to reduce pain, and improving clinical outcomes (Roger et al., 2002). On this point, Lederman (2017) argues that manual therapy techniques should be viewed as a vehicle to deliver touch effects, which can have a positive influence on the sense of “self,” wellbeing, and body image, as well as a profound calming and soothing influence on the individual.

This article will focus on therapeutic touch as a synonym for human touch-based interventions used in MSK care. Hands-on care relies on three crucial dimensions of touch, i.e., analgesic, somatoperceptual and affective (Geri et al., 2019). Arguably, these dimensions fall broadly into two categories – discriminatory and affective touch (McGlone et al., 2014, 2017). Most people understand the sense of touch as a discriminative sense, enabling us to detect, for example, a fly landing on our face, or the texture of the surface being manipulated – discriminatory or “fast touch” relies on large myelinated (Aβ) afferents which project primarily to the somatosensory cortex (McGlone et al., 2014). Discriminatory touch has been traditionally associated with the observed modulatory effects of manual therapy and therapeutic touch on the central and autonomic nervous system (i.e., Mancini et al., 2014; Geri et al., 2019).

On the other hand, affective touch is the “slow” touch system that is dependent on a system of unmyelinated low threshold mechanosensitive c-fibres called c-tactile (CT) afferents, which project mainly to emotion-related paralimbic cortical systems (insular cortex), the posterior superior temporal sulcus (pSTS) and the medial prefrontal cortex (mPFC)/dorsoanterior cingulate cortex (dACC) (McGlone et al., 2014). CT afferents cannot provide helpful discriminative information due to the slow conduction velocity of C-fibres (McGlone et al., 2014, 2017). The CT afferent system plays a crucial role in providing or supporting emotional, hormonal, and behavioural responses to skin-to-skin contact with conspecifics (McGlone et al., 2014). Therefore, affective touch is associated with tactile stimuli with a hedonic and often emotional component, encompassing aspects of reward and significance in social communications (Morrison et al., 2010; Morrison, 2016a).

The insular cortex integrates the sensory and emotional systems creating an interoceptive modality. Interception is defined as the perception of internal physiological states within an individual’s body and the sensations of an external stimulus such as touch or pain (Craig, 2002; Panagiotopoulou et al., 2017). CT and Aβ afferents are required for the complete feeling of pleasant touch on the hairy skin (McGlone et al., 2014). Moreover, the insular cortex’s activation during therapeutic touch affects the modulation of interoceptive precision (attention) and contributes to the pleasant feelings associated with therapeutic touch. Crucially, the modulation of interoceptive precision (attention) by the insular cortex also alters body awareness, autonomy, and sense of self, updating interoceptive beliefs associated with the use of therapeutic touch while also being innervated by the transcutaneous auricular vagus nerve (Craig, 2008; Crucianelli et al., 2013, 2018; Ainley et al., 2016; Park et al., 2017; Bradran et al., 2018; Paciorek and Skora, 2020).

The vagus nerve can also provide inhibitory inputs to the heart, which are crucial for self-regulation, as proposed through the neurovisceral integration model (NVIM) (Park and Thayer, 2014). The NVIM suggests that an individual’s ability to adapt to its environment depends on the physiological flexibility within the different hierarchical levels and attractor basins of the central autonomic network (CAN), in which the vagus nerve and vagal control are crucial to the feedback mechanism (Thayer and Friedman, 2002; Condy et al., 2020).

The clinical benefits of therapeutic touch in MSK care are likely to depend on both discriminatory, “fast” touch and affective, “slow” touch (McGlone et al., 2017). Therapeutic touch has shown to be effective for pain relief, reducing heart rate and systolic blood pressure in patients with persistent non-malignant pain and even in those with other comorbidities such as breast cancer (Billhult et al., 2009; Olufade et al., 2015; Pinheiro da Silva et al., 2019). Moreover, therapeutic touch in the form of massage initiates relaxation, sense of safety, reduces fear-avoidance and physiological markers of stress, i.e., cortisol and heart rate through deactivating the threat of noxious stimulus and possibly initiating autonomic regulation, particularly in chronic LBP, arthritis, fractures, and pain conditions (Hernandez-Reif et al., 2004; Billhult et al., 2009; Maratos et al., 2017; Miciak et al., 2019).

Touch with the intention to care for another, such as in a clinical environment, provides better relief from physical and emotional distress than self-care by decreasing the activation of pain-related regions, particularly the dorsal Anterior Cingulate Cortex (ACC) and the Anterior Insular Cortex (AIC) (Shamay-Tsoory and Eisenberger, 2021). The insula is a crucial region in pain processing, and developing evidence indicates that the insula encodes the magnitude of an unexpected outcome – unsigned prediction errors (Horing and Büchel, 2022). Prediction errors occur in events where there is a mismatch between an expected and actual event or sensory signal (Rossettini et al., 2022). Prediction errors are crucial in updating a generative model and, therefore, in driving learning (Parr et al., 2022). Recent evidence indicates that a misrepresentation of learning relevant prediction errors in the insula is likely an underlying factor in persistent pain (Horing and Büchel, 2022).

Moreover, the insular cortex is also part of the Salience and Default Networks that assist with regulating the nervous, immune, and neuroendocrine systems, which play a crucial role in regulating allostasis (Atzil et al., 2018). Allostasis is the adaptive anticipatory process of, which delivers the right kind of context for achieving homoeostatic balance; namely, to achieve the primary goal of maintaining a stable bodily state through a series of physiological (i.e., autonomic) or behavioural (i.e., sensorimotor) closed feedback loops (i.e., reflexes). Crucially, the primary function of the nervous system is to manage allostasis, i.e., predicting the physiological needs for survival (Barrett, 2020; Esteves et al., 2022). Importantly, in the context of MSK care, allostatic load, and overload – the cumulative burden of chronic stress and life events – has been linked to poorer health outcomes, including pain, depression, anxiety and MSK disorders (see Guidi et al., 2021, for a recent review). The role of the insular cortex in allostatic regulation is also crucial in building trust and attachment through a collaborative relationship to secure joint attention, with synchronisation allowing empathy and acknowledging it as being rewarding, and thus decreasing pain (Barrett and Fleming, 2011; Tomasello et al., 2012; Shamay-Tsoory and Eisenberger, 2021; McParlin et al., 2022). Moreover, the insular cortex is also central to oxytocinergic modulation, encouraging and modulating the development of bonding, trust, and processing of therapeutic touch, which can also contribute to building a robust TA (Morrison, 2016a; Fotopoulou et al., 2022; McParlin et al., 2022). Therefore, it is crucial that clinicians understand the role of allostatic load and its regulation on patient care. Arguably, therapeutic touch can play a key role in allostatic regulation and achieving embodied predictions regarding social attachments, achieving balance amongst all bodily systems needed for survival and physiological co-regulation with others, through caregiving touch in specific social interactions (see Fotopoulou et al., 2022, for a review).

The therapeutic alliance (TA) is referred to as the collaborative relationship or working alliance between the patient and Health Care provider (HCP) and is necessary for establishing a positive rapport and trust, ensuring patient satisfaction, and achieving positive clinical outcomes following treatment, particularly in patients with persistent MSK pain (Taylor et al., 2015; Kinney et al., 2018; Miciak et al., 2018; McCabe et al., 2021b). This article will refer to an HCP as an orthopaedic manual physical therapy practitioner specialising in managing neuro-musculoskeletal conditions using manual techniques and therapeutic exercises, including physiotherapists, osteopaths, chiropractors, and massage therapists (International Federation of Orthopaedic Manual Physical Therapists [IFOMPT], 2004).

The TA is centred around the tripartite elements of a working alliance described by Bordin (1979), including the agreement on goals, agreement on a task, and the development of attachment bonds, which are the foundation for a successful TA (Rossettini et al., 2020). An HCP who creates a successful person-centred TA can gain the trust of anxious and sceptical patients. Moreover, in patients, particularly those with chronic low back pain, a good TA can result in positive clinical outcomes for both physical and mental capacities, overall patient satisfaction, and quality of life (Ferreira et al., 2013; Fuentes et al., 2014; Taccolini Manzoni et al., 2018; Rossettini et al., 2020). To form a collaborative agreement needed for a robust TA, one needs to adapt their personal opinions to be in tune with other individuals. We argue that this can be formulated as active inference.

Active Inference (AI) is a “first principles” approach to understanding sentient behaviour—perception, planning and action in terms of probabilistic inference—framed as a single imperative to minimise free energy (Esteves et al., 2022; Parr et al., 2022). AI enables an organism or an agent to adjust to its environment to fit its expectations and therefore construct its niche (Bruineberg et al., 2018; Constant et al., 2018; Esteves et al., 2022). In particular, agents build shared expectations through engagement with everyday social and material affordances, allowing adaptive niche construction by, for example, thinking through other minds (Laland et al., 2001; Boyd et al., 2011; Veissière et al., 2020; Esteves et al., 2022). Taken together, AI reflects the natural inclination of all living organisms to regulate themselves – within their environment – by optimising their internal world model in a series of self-fulfilling action-perception cycles. Subsequently, it will minimise “surprise” by becoming in tune with others (Bruineberg et al., 2018; Constant et al., 2018; Vasil et al., 2020). Surprise, in this context, can be read as a prediction error or, mathematically, the implausibility (i.e., negative log-likelihood) of some sensory outcome, given a world model or narrative that would predict that outcome.

The NVIM can also explain an individual’s ability to adjust to their context-specific niche as it proposes the flexibility to adapt to an external environment is dependent on weighting attributed to each prior as well as the hierarchical model proposed for vagal control and corresponding attractor basins within the CAN (Smith et al., 2017). Each layer of the neurovisceral integration loop transfers prediction errors from lower to higher sensory levels, which could involve the overall state-space of the CAN, which is partly vagally mediated (Thayer et al., 2009; Park and Thayer, 2014). Through AI, the hierarchical nature-space of the CAN allows for the effective minimisation and adjustment of prediction errors through attention to determine which level and output of the CAN is appropriate for regulating both visceral and skeletal motor physiology (Thayer and Lane, 2000; Smith et al., 2017).

Active inference has been applied to therapeutic practices as a model to explain symptomatic presentations of predicted sensory information compared to current sensory stimulus, with persistent pain being a overestimate of prior beliefs compared to current and often less noxious sensory stimulus, also called a failure of inference (Henningsen et al., 2018; Pezzulo et al., 2019; Bohlen et al., 2021). Inappropriate estimation of low precision for maladaptive beliefs could be due to higher CAN levels for the specific prior, inappropriately lower precision with higher weighting at lower hierarchical levels (Hechler et al., 2016; Smith et al., 2017; McParlin et al., 2022). Moreover, difficulty in updating maladaptive embedded priors and prediction errors is also found with sustained autonomic response reflected in the creation of more set points across the hierarchical levels of the CAN, with individuals getting “stuck” in the maladaptive attractor basin.

Active inference has also been applied to TA, patient-centred care and biobehavioural synchrony in combination with touch as a sensory stimulus to intentionally share, update and generate new prediction errors in an attempt to reduce symptoms, restore allostasis, self-regulation and agency (Bohlen et al., 2021; Esteves et al., 2022; McParlin et al., 2022). Cooperative communication is the intentional interaction between individuals to align their mental states (Tomasello, 2019). Therefore, gathering evidence to endorse an individual’s beliefs by synchronising their mental states with another’s mental states in a shared situation strengthens the ability for cooperative communications and an effective TA (Vasil et al., 2020). Arguably, therapeutic touch can be an efficient way to achieve attunement and collaborative interpersonal relationships and biobehavioural synchrony and alignment with others (Csibra, 2010; McParlin et al., 2022).

We propose that therapeutic touch in MSK care can help develop and enhance cooperative communications and strengthen the TA between the patient and the HCP, while restoring homoeostasis and allostatic balance to resolve the patient’s clinical problem. A predictable and positive relationship between the HCP and a positive TA is considered more influential than individual attachment preferences, including any pre-existing anxieties when developing a new relationship with another individual (Taylor et al., 2015). In clinical practice, collaborative interpersonal relationships also contribute to patient control and self-efficacy by encouraging active participation and adherence to exercises (Babatunde et al., 2017; Kinney et al., 2018). Therefore, touch could help develop a successful collaborative and therapeutic relationship contributing to the good clinical benefits of a positive TA (Miciak et al., 2019). The remaining sections unpack the clinical application of touch in MSK care, through the lens of active inference.

## Person-Centred Approach: Recognising Priors

A person-centred approach to treatment is crucial for achieving shared attention and cooperative communication, producing positive outcomes in managing MSK conditions (Shannon and Hillsdon, 2007; Kim and Park, 2017; Hutting et al., 2022). Person-centred care is structured around the individual’s specific needs and preferences, enabling confidence-building and the development of a robust TA, particularly in rheumatoid conditions and chronic LBP (Haugli et al., 2004; Lærum et al., 2006; Reynolds, 2009; Barbari et al., 2020). Person-centred occurs when, for example, an HCP adapts a standard rehabilitation protocol to make it more time-efficient or acknowledges that a single mother or individual with financial constraints may not complete the protocol in its entirety (Miciak et al., 2019). On this point, patients with LBP and other MSK conditions express appreciation and a stronger bond with their HCP when personal adjustments are made (Del Baño-Aledo et al., 2014; GMC, 2020). A person-centred approach underpinned by a robust TA provides opportunities for discussing personal beliefs and fears. Moreover, it improves adherence to treatment advice and rehabilitation in patients with LBP, shoulder, and hip osteoarthritis (Lequerica et al., 2009; Hall et al., 2010; Cheung and Soundy, 2021).

Bayesian beliefs are the – sub-personal war propositional – dispositions and narratives that guide a person’s choices and behaviour, based on their prior experiences and cultural legacy. Bayesian beliefs are important adaptive priors to consider in the context of patient care: they are evolutionary, subpersonal, socially and culturally inherited (Badcock et al., 2019a,b). These priors will have been developed over time to create more adaptive and veridical predictions of outcomes in the lived, prosocial world (Ramstead et al., 2018; Badcock et al., 2019a). It has been suggested that empirical assumptions of Bayesian beliefs with personal priors and sensory input [*s* = *g*(*η,a*) + ω], including touch accounting for an individual’s internal states (μ) and affecting how an individual chooses to interact with their external world (Ramstead et al., 2018; Badcock et al., 2019a). The external world can be empirically quantified through equations of motion [η = *f*(*η,a*) + ω], specifying the hidden dynamics of the world accounting for random fluctuations (ω) of internal and external states synergistically attempting to minimise free energy. Free energy is the probability of hidden environmental causes [*q*(*η:μ*)] and sensory inputs determined by the individual’s internal states (*F* = *Energy-entropy*). Free energy can be minimised by increasing the accuracy of sensory data, i.e., picking more reliable data based on priors (*F* = *complexity–Accuracy*) or synchronising with more experienced individuals, i.e., HCP allowing the variation of free energy to impose tighter bonds reducing surprise from sensory or physiological states (*F* = *divergence* + *surprise*).

We use Bayesian belief to denote a specific viewpoint or expectation encoded by neural representations in higher cortical regions that send predictions to hierarchically lower levels (Friston and Frith, 2015; Vasil et al., 2020). These descending predictions can then be compared with expectations at lower levels to form a prediction error. Subsequently, the prediction error is then passed back to high levels to revise Bayesian beliefs – and thereby instantiate a process of Bayesian belief updating that enables the patient to, literally, make sense of their world. These priors regulate and influence information processing within treatment sessions and contextualise any approach any treatment issues (Hasson et al., 2015). HCPs must understand their patients’ perspectives, goals, and priors by mentalising higher-order cognitive processes. This facilitates a common ground – or shared narrative – and a beneficial clinical outcome (Frith and Frith, 2006; Miciak et al., 2019). Therefore, the development of a robust TA is dependent on the HCP’s ability to identify, accept and acknowledge an individual’s unique priors, context, and expectations. Consequently, HCP can gain trust and therefore develop a personalised patient-centred treatment.

Recognising priors is particularly crucial in chronic pain due to its complexity and multifactorial nature when compared with acute pain. In the context of chronic LBP, social factors are as significant as physical factors in the patients’ pain experience (Hill and Fritz, 2011; Louw and Puentedura, 2013). To this end, patients with chronic non-malignant pain and other MSK disorders consider being listened to, believed and viewed more than just their symptoms or condition to be crucial to their quality of care (Bordin, 1979; Lærum et al., 2006; Clark, 2013; Wilson et al., 2017; Søndenå et al., 2020). This genuine interest in the person beyond their clinical condition can put patients at ease, allowing them to relax and be more comfortable with their HCP (Miciak et al., 2018). Arguably, in the context of MSK care, an individual’s preferences and responses to therapeutic touch are also modulated by the same factors that affect a therapeutic relationship, including cultural and social priors (Sorokowska et al., 2021). Therefore, a person-centred approach with the treatment specifically tailored to the individual is essential in all aspects of a clinical encounter.

Prior beliefs regarding pain and a range of MSK disorders are not always conducive to patient recovery (Rossettini and Testa, 2018). Levels of maladaptive (i.e., false) beliefs and accompanying attitudes are high among patients with chronic LBP (Christe et al., 2021). These include catastrophising and fear avoidance, leading to higher levels of pain, disability and poorer clinical outcomes. One pernicious aspect of these “false” beliefs is that they preclude actively seeking evidence that would revise them (e.g., “I can’t move because it would hurt”). These maladaptive beliefs are often linked to unaffectionate HCPs (Wertli et al., 2014a,b; Cheung and Soundy, 2021). Therefore, personalised, attentive care targetting and updating these beliefs are significant in aiding recovery (Main et al., 2010; Linton and Shaw, 2011).

## Application of Active Inference to Touch and Therapeutic Alliance

In active inference, our choices and behaviour determine the sensory data we use to make inferences, including touch. This includes overt action such as “palpation” of the visual world through saccadic eye movements or touch, to covert action such as the deployment of attention, or precision to newsworthy sensory information (Seth and Friston, 2016; Parr and Friston, 2017; Parr et al., 2018; Sterzer et al., 2018; Smith et al., 2019; Limanowski et al., 2020). In AI’s predictive processing (i.e., predictive coding) formulations, the newsworthy information corresponds to prediction errors, namely, the difference between our predictions based on our prior beliefs and what we actually sensed. These prediction errors update beliefs when and only when they are considered dependable or precise (Kok et al., 2012; Brown et al., 2013; Veissière et al., 2019; Limanowski et al., 2020). However, it is essential to note that the reliability or precision given to prediction errors can be irrational and maladaptive, particularly in persistent pain sufferers.

Physiologically, this corresponds to increasing the synaptic gain of various neuronal populations encoding prediction errors (Vossel et al., 2014; Auksztulewicz and Friston, 2015). Psychologically, this can be thought of as sensory attenuation or selective attention, depending upon whether the precision is inferred to be high or low (Feldman and Friston, 2010; Adams et al., 2013; Moran et al., 2013; Limanowski, 2017). In short, “precision” describes the reliability or “trustworthiness” afforded prediction errors. The consequent precision weighting of prediction errors may depend upon epistemic trust and a mutual narrative (Fonagy and Allison, 2014). This also implicates the building of trust essential to a robust TA.

In addition to increasing the precision via selective attention, the attenuation of certain prediction errors (via reducing their precision) is necessary to ignore certain sensations. Sensory attenuation is crucial in this context because sensory attenuation is necessary to act upon the world. In other words, to execute a predicted or intended movement, it is essential to ignore sensory evidence that the movement has not yet been initiated. Anecdotally, sensory attenuation may explain why “rubbing one’s neck” attenuates nociceptive signals that would otherwise be explained by the prior belief or experience “I have a neck pain.” This maladaptive overestimate in the weighting of sensory information is commonly found in chronic pain patients whose overly precise prior beliefs predict their pain symptoms before the noxious sensory information actually occurs (Hechler et al., 2016; Van den Bergh et al., 2017; Kube et al., 2020). Furthermore, alternating augmentation and attenuation of sensory precision may be crucial in dyadic interactions, in the sense that it underwrites “turn taking” in communication (i.e., Wilson and Wilson, 2005; Ghazanfar and Takahashi, 2014; Friston and Frith, 2015). In other words, listening and speaking when establishing a shared narrative requires a reciprocal attenuation and augmentation of the shared sensory modality, commonly found in therapeutic touch and MSK care.

Therapeutic touch with a solid affective component may be a particularly potent interoceptive modality for this kind of communication, allowing the updating of priors at two levels. Firstly, it establishes a sensory modality of exchange, which provides sensory evidence that “you are like me.” This is a crucial inference that enables the use of the same (shared) narrative to finesse predictions of “self” and “other.” The notion of a shared narrative or generative model of interpersonal exchange may be an essential aspect of the TA, especially regarding the agreement on goals and treatment plans. Secondly, the particular effect of therapeutic touch may draw attention (i.e., precision) to the sensory levels of hierarchical inference, thereby reducing the relative precision or commitment to higher-level prior beliefs. A reduction or relaxation of the precision and reliance of prior beliefs is generally thought to be a fundamental prerequisite for belief updating in a therapeutic setting (please see Ainley et al., 2012, 2016; Duquette, 2017, 2020; Carhart-Harris and Friston, 2019) for treatments of precision and interception in psychotherapy.

Moreover, many aspects of psychotherapy have played a role in the research and practice of manual therapies, particularly in the care of patients with chronic LBP and other MSK disorders (McCabe et al., 2021a; Hutting et al., 2022). In short, synchronous exchange between individuals enables more accurate inference of the partner’s subsequent actions and coordinates joint attention and implicit precision weighting. Through the hierarchically organised model it helps to facilitate belief updating under a shared narrative through equal collaboration and (mutual) epistemic trust that may underwrite the TA, creating symmetrical coupling and synchronisation (Moran et al., 2013; Fonagy and Allison, 2014; Hasson and Frith, 2016). In other words, it assists HCPs in navigating and implementing the most effective communication strategies while minimising prediction errors and uncertainty necessary to establish an optimal therapeutic alliance and maintain homoeostasis which can be done through therapeutic touch and resulting in biobehavioural synchrony with the other individual (Duquette, 2017, 2020; Carhart-Harris and Friston, 2019).

## Biobehavioural Synchrony Initiated Through Touch

Touch can often provide the sensory evidence to help conclude that “you are like me” through stimulating biobehavioural synchrony in which different individuals harmonise their biological and behavioural processes during social interaction (Feldman, 2012, 2017; Koole and Tschacher, 2016; Tschacher et al., 2017). Therapeutic touch during a massage in healthy individuals affects the modulation of psychological and neuroendocrine function through the stimulation of mechanical receptors, which share the same innervation as vagal afferent fibres, which are subsequently involved in the regulation of the autonomic nervous system, thus causing decreased heart rate, blood pressure and stress (Diego and Field, 2009). The processes involve coupling physiological and behavioural processes across the four systems of matching non-verbal behaviour, coupling heart rhythms, respiratory, autonomics, brain to brain synchrony, motor movements, and coordinated oxytocin, dopamine, and cortisol (Feldman, 2017; Goldstein et al., 2017).

Biobehavioural synchrony can be evident immediately with therapeutic touch by increased heart rate variability, decreased anxiety, anger and pain in patients with chronic tension-type headaches (Toro-Velasco et al., 2009). Hands-on techniques can also influence the autonomic nervous system and cause bidirectional release of neurotransmitters such as oxytocin between the HCP and the patient (Uvnäs-Moberg, 2004; Cerritelli et al., 2020a). This supports the argument that therapeutic touch can help in the embodied transfer of an individual’s parasympathetic regulation to another (Van Puyvelde et al., 2019). The exact mechanism behind biobehavioural synchrony varies depending on the type of bond; for example, patient-practitioner synchrony would be different from a romantic partnership. The threat level also plays a role with higher synchronisation of their actions and posture even if they are not aware of it in situations of stress or threat, i.e., a painful MSK injury (Goldstein et al., 2017). The release of oxytocin is dependent on the level of attachment which determines the coupling of oxytocin and dopamine before being combined in the subcortical to cortical networks involved in reward, embodiment and mentalisation (Feldman, 2017). Consequently, when consolidating interpersonal relationships and achieving biobehavioural synchrony in social situations, synchronous activation of the temporoparietal regions and heart rate is considered a key component to achieving brain to brain alpha coupling (Shamay-Tsoory et al., 2019). The synchrony is often visible within a therapeutic relationship in the clinician’s temporoparietal, prefrontal, and AIC activation, indicating social cognition and mirroring with the patients’ dyads (Ellingsen et al., 2020). The right prefrontal cortex becomes activated when one individual is perceived as the leader, inhibiting another individual’s self-representation (Fairhurst et al., 2014a,b).

## Developing and Updating Priors Through Touch

Socio-affective touch can adjust the relative importance and response of noxious stimulus in the AIC and cingulate cortices (Krahé et al., 2013; Von Mohr et al., 2018). Touch can create bio-feedback loops that help develop and learn to develop new or update less precise priors by altering the setpoint in crucial survival demands. Touch can create bio-feedback loops that help develop and learn to develop new or update less precise priors by altering the setpoint in crucial survival demands (Rossettini et al., 2022). This viewpoint was initially proposed by Sterling and Eyer (1988) and has been expanding to include the integration of active inference to interception, regulation of homoeostasis and allostasis, all based on recent advances in anatomical knowledge, empirical models, and computational neuroscience (Stephan et al., 2016; Fotopoulou et al., 2022). When developing a prior, brain regions such as the AIC, Anterior cingulate cortex (ACC), orbitofrontal cortex (OFC), and subgenual cortex (SGC) will generate allostatic predictions to embody a generative model of the current input (Pezzulo et al., 2015; Corcoran et al., 2020; Tschantz et al., 2022). We would argue that one can include therapeutic touch in predicting the bodies currently and future state. These brain regions will receive prediction errors about interception from the posterior and mid insula. Other allostatic top-down predictions enable them to modulate the current homoeostatic beliefs found in the subcortical regions fulfilled by reflex arcs in areas such as the hypothalamus and brainstem (Stephan et al., 2016; Fotopoulou et al., 2022).

The descending projections from the AIC, ACC, OFC, and SGC could relay the individuals top-down predictions to the posterior and mid insular cortex to compare them with bottom-up sensory afferents to form the prediction errors necessary for precise allostatic responses to the current situation. A clinical example of unpredicted sensory stimulation – incorporating therapeutic touch – is through guided exercises or passive movements. The HCP will help the patient complete a series of movements they may previously find painful or were too nervous about doing independently. Moreover, it allows the patient to observe and gain tactile sensory information, actively. They can do the movement, even if the HCP is physically doing the action for them, such as a passive joint examination. All areas mentioned above connect to the granular layer IV of the primary interoceptive insular cortex allowing the integrative modulation of homoeostatic beliefs (Sterling and Eyer, 1988; Fotopoulou et al., 2022). These assumptions are sustained and expanded upon from the recognised anatomical and hierarchical structure of laminar patterns in Macaque monkeys (Li et al., 2017). Neuroimaging studies have discovered projections between the AIC, ACC, OFC, and SGC regions during the activation of CT fibres suggesting further interconnectivity (McGlone et al., 2012; López-Solà et al., 2019; Shamay-Tsoory and Eisenberger, 2021).

**FIGURE 1:**
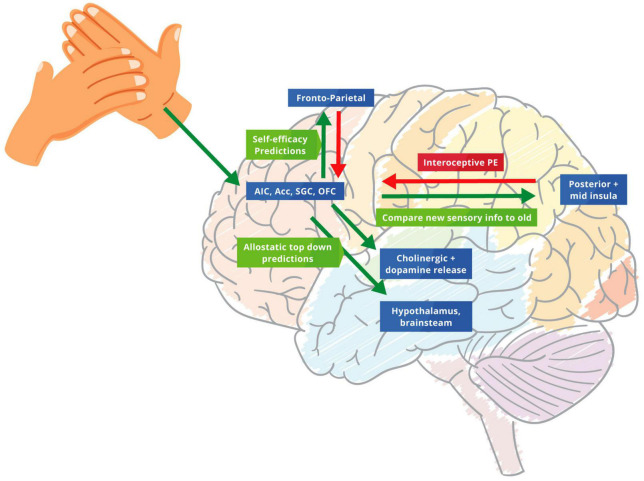
The biofeedback loop of therapeutic touch. Green arrows: predictions errors; Red arrows: predictions. Touch biofeedback loop adapted from Sterling and Eyer (1988) and Fotopoulou et al. (2022) to help modify and adjust the importance of priors through new allostatic predictions from therapeutic touch. We suggest that the AIC, ACC, SGC, and OFC create allostatic therapeutic touch predictions that underwrite interceptive prediction errors in the posterior and mid insula. These predictions modulate homoeostatic beliefs in the hypothalamus and brainstem. Descending projections from the AIC, ACC, OFC, and SGC replay top-down predictions to the mid and posterior insula to protect the new bottom-up stimulus of therapeutic touch. The ensuing prediction errors are then sent to the frontoparietal network that revises prior beliefs and future allostatic predictions, identifying more precise and newsworthy prediction errors for this specific situation for the future.

Ascending prediction errors target the frontoparietal network, thereby revising descending self-efficacy automatic sensory predictions to help update future allostatic predictions. This process creates a better generative model of the “self” that is increasingly precise and aligned within the current situation, potentially de-threatening the noxious stimuli (Fotopoulou and Tsakiris, 2017; Owens et al., 2018). In essence, the HCP introduces a “surprise” to the system through the tactile sensory stimulus, which is part of the hands-on techniques. This “surprise” enables a functional belief updating that revises prior beliefs that underwrite the patients “illness.” Subsequently, this enables the patient to increase the weighting of interoceptive prediction errors about the current state of the “self” ascending to higher hierarchical levels (Hechler et al., 2016). Moreover, this process helps predict the right level of precision to be deployed in certain situations. This process of predicting precision appears to be mediated by cholinergic, dopamine and other neuromodulators – to optimise attentional set (Atzil et al., 2018; Cox and Witten, 2019; Crucianelli et al., 2019; Nguyen et al., 2021). This contextualisation of predictive processing rests on top-down predictions occurring before the consequences of touching or being touched (Friston et al., 2015; Figure 1).

## The Effect of Touch in the Management of Chronic Musculoskeletal Pain

There is substantial evidence showing that the expectation and prediction of severe pain alone in chronic MSK pain can increase the pain felt and cause symptoms before the trigger occurs (Rossettini et al., 2018). This is observed in patients with fibromyalgia and arthritis who often predict the source or trigger to their increase in symptoms, falsely interpreting a non-noxious stimulus as painful, in order to fulfil their belief of recurrent, persistent pain (Vlaeyen and Linton, 2000; Brown et al., 2013; Henningsen et al., 2018). In these instances, it can be argued that therapeutic touch can help re-establish their misinterpreted bodily sensations or develop new explanations for their symptoms (Xu et al., 2014; Barrett and Simmons, 2015). In the absence of such therapeutic interventions, pain sufferers can have a persistent and self-fulfilling “failure of inference” (Tracey, 2010; Di Lernia et al., 2016; Hechler et al., 2016). Subsequently, persistent pain patients may be cognitively immunised to updating or changing their priors, so that they discount the interactions with the HCP and anticipate that the clinical encounter will be irrelevant; thereby reaffirming their maladaptive priors, even before they present for treatment. This can lead to the HCP being regarded as an imprecise source of sensory information, and their sensory input and stimulation are dismissed (Fonagy and Allison, 2014).

**FIGURE 2:**
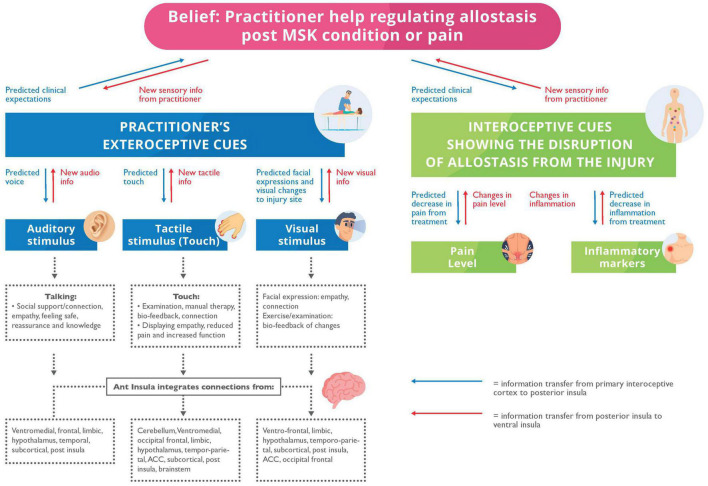
A mode to describe the allostatic regulation by a Health Care Provider (HCP). Blue arrows: The predicted expectations and priors transferring information from the primary interoceptive cortex to posterior insula. Red arrows: New sensory information from the posterior insula to the ventral insular. Exteroceptive: We propose that the patient has predicted expectations for the clinical appointment and predictions about the HCP’s stimuli. We offer putative multisensory mechanisms, i.e., auditory, touch and visual – for allostatic regulation from the HCP. They help display support, empathy, reassurance, and improved clinical function; all are originating from the anterior insula. The anterior insula integrates this multisensory input for allostatic regulation from the cerebellum, ventromedial, limbic, temporoparietal, subcortical, posterior insular, occipital frontal, temporal cortices, anterior cingulate cortex, hypothalamus, and brainstem. Interoceptive: The patient will predict or expect clinical expectations of interoception because of treatment including reduced pain and inflammation which we propose originate from new tactile and proprioception (i.e., haptic) stimulation from the HCP.

The tactile and proprioceptive stimulation from handsoncare conveys interoceptive and affective information that adjusts the processing of sensory information that undergirds symptoms (McGlone et al., 2014; D’Alessandro et al., 2016; Quadt et al., 2018; Bohlen et al., 2021) (see Figure 2). Cerritelli et al. (2020b) endorse this view by finding that osteopathic treatment increases the interoceptive accuracy of patients with chronic LBP – with accompanying decreases in the blood oxygenation level dependant (BOLD) levels in the bilateral insula, ACC, left striatum and right middle frontal gyrus; brain regions implicated in interception. This suggests that the brain becomes sensitised to touch as a precise and newsworthy sensory modality (Scalabrini et al., 2019). Additionally, this modification in body perception, awareness of pain levels, and location through touch is specific to patients – with conditions like LBP – who cannot visualise the source of symptoms and may therefore imagine the symptomatic area being more extensive than it is (Nishigami et al., 2015; Puentedura and Flynn, 2016).

Therapeutic touch – supported appropriate and non-nocebic language – can create new interoceptive biofeedback loops to promote reassurance, communication, and joint attention over the injury: for example, by commenting, “This muscle feels tense, or the joint is stiff. Do you agree?” or showing that pressing the injury while painful is not making the injury worse (Rossettini et al., 2018). By physically exploring the area, it will provide insight and a link to the “internal situation” beneath (Harman et al., 2011). Arguably, the body is the physical manifestation of our hidden thoughts or world, and HCPs can uncover hidden beliefs through physical examination (Thornquist, 2001). In short, changing the patient’s prior beliefs that she is a patient with “chronic pain” into a belief that she is “recovering from a chronic pain condition” enables the patient to ignore and reinterpret interoceptive signals, emulating the attenuation of sensory (interoceptive) precision (Hoskin et al., 2019; Gerrans, 2020; Seymour and Mancini, 2020). Nonetheless, it is more difficult to form new priors if entrenched through self-reference (Siu and Humphreys, 2015). Despite the HCP’s best intentions, it is possible that over-precise priors will cause a lack of attention – and therefore effectiveness – to hands-on care and interventions of the HCP.

Crucially, not only should the HCP consider the patient’s priors, but they must be aware of their own priors that may influence and modulate interpersonal behaviour (Horton et al., 2021). If the HCP is aligned with their patients, some disclosure of the HCP’s past can frequently deepen the connection and help the patient feel that they are just like them (Meltzoff, 2007; Miciak et al., 2019). While not all HCPs are comfortable with this, revealing some of their inner personal experiences can help increase the TA, trust, and bond between patient and HCP, thereby increasing the common ground and synchrony. In patients with MSK disorders, if the HCP is more open, approachable and expresses themselves more freely and personally, it can help patients to freely express their feelings, increasing trust, which can extend to taking more of a leap of faith with their physical symptoms and injuries (Miciak et al., 2018).

## Joint Action-Perception Cycles Within Treatment

**FIGURE 3:**
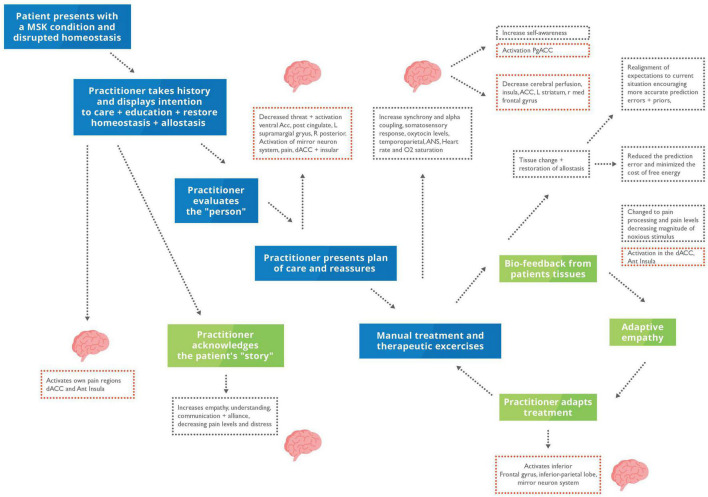
Putative model homoeostatic regulation in musculoskeletal (MSK) care during a clinical encounter. Blue box, clinical presentation and practitioner’s actions; Green box, interoceptive metacognitions; Red dotted box, changes in brain activity; Black dotted box, changes to interoception and mental states. The patient will present with disrupted homoeostasis because of their clinical condition. During a clinical encounter, the HCP will take a case history, examine their patient, and discuss their condition and care plan. The HCP will reassure, educate, understand, acknowledge, and empathise with their patient. We propose that hands-on care which is underpinned by a robust therapeutic alliance and communication can putatively restore homoeostasis through increasing biobehavioural synchrony and active inference – thus updating the patient’s generative model.

The TA principles revolve around an agreement between patient and HCP on the treatment plan and goals of the patient, which is particularly important for managing many MSK conditions, including LBP. With a closer bond, the patient may perceive others to be more similar and isomorphic with themselves, than an inanimate replica (Scalabrini et al., 2019). This increase in synchrony may also contribute to teamwork and interpersonal collaboration in manual therapy, which is developed through collaboratively agreeing on a set goal (Miciak et al., 2019; Brun-Cottan et al., 2020) (See Figure 3 for the application to MSK clinical encounters). AI and cooperative communication both suggest that the alignment of mental states with another helps to minimise supplies and resolve uncertainty (Constant et al., 2019; Tomasello, 2019). Similarly, the Social Baseline Theory suggests that humans are built to be social and work with familiar individuals in uncertain situations or when approaching problems (Coan and Maresh, 2014; Beckes and Sbarra, 2022).

**FIGURE 4:**
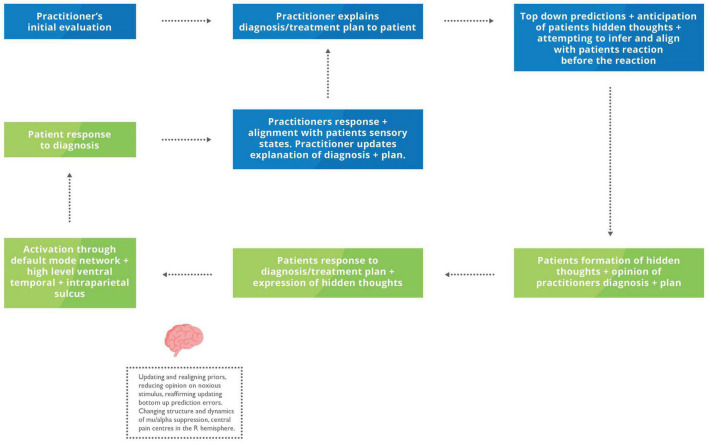
Proposed feedback loop during an explanation of the problem and plan of care. Blue box, HCP inner mental states and actions; Green box, Patient internal mental states and actions. The HCP conducts an initial examination, often using verbal and non-verbal communication – including touch to reach a summary of their evaluation and plan of care. As the practitioner communicates the outline of their assessment, they will start to mentalise their patient’s reactions and hidden throughs. The patient may begin developing hidden thoughts regarding their diagnosis before verbalising them. Based on their patient’s responses, the HCP may modify their previous explanation to achieve a more robust and collaborative description considering the patient’s reactions.

Individuals generate and modify their own feedback loops to achieve the end goal of aligning their mental states with others (Friston and Frith, 2015). Universally, this involves action and perception loops that reliably connect the two distinct policies until they become entwined and produce a shared narrative (Friston and Frith, 2015). Social collaboration to achieve and maintain allostatic needs helps decrease the risk of death and injury due to the shared goal, trust, and familiarity between the groups’ individuals (Vasil et al., 2020). Touch is often considered a standard modality to achieve this through socio-affective regulation involving cognitive, metacognitive process and embodiment (Roberts and Bucksey, 2007; Aureli and Presaghi, 2010; Coan and Maresh, 2014; Fotopoulou et al., 2022). Examples of the socio-affective regulation include positive changes in immunity, inflammation, and neuroendocrine function by decreasing stress, allostatic load and subsequently effort to maintain homoeostasis in critically ill or multifactorial conditions such as chronic pain that have all been achieved through touch (Papathanassoglou and Mpouzika, 2012; Coan and Maresh, 2014; Morrison, 2016a; Kerr et al., 2019). The clinical appointment is one example of a coupled action-perception cycle and social collaboration; both in the agreement of the diagnosis and within the hands-on therapeutic intervention. That patient will present with pain or abnormal physiology, resulting in an allostatic overload and seeking help from the HCP to re-establish her allostatic balance (McEwen and Wingfield, 2003; Beckes and Coan, 2011; Miciak et al., 2019; Guidi et al., 2021; Vasil et al., 2020) (see Figure 4).

With explanation of the diagnosis – and with the use of therapeutic touch – the HCP can establish mutual synchrony by continuously inferring the patient’s reaction, pain and symptoms and adjusting their explanation or touch to align with the patient’s sensory states. For example, if the HCP infers the tactile pressure is excessive compared to the patient’s pain or physiological response, they will adjust their predictions and decrease the pressure and its subsequent effects and vice versa if not strong enough. Additionally, it has been shown that different massage strengths, light vs. moderate, have slightly different effects, with moderate massage increasing parasympathetic and light increasing the sympathetic nervous system (Lindgren et al., 2010). The HCP will continue to constantly adjust their touch until they are satisfied with the change in tactile feedback from the technique, i.e., increased movement in the joint space of exchange and restoring the patient’s homoeostasis. Restoring homoeostasis and hopefully decreasing the symptoms can be considered a reduction in the prediction error (a.k.a., free energy) to regulate allostasis (Peters et al., 2017; Atzil et al., 2018; Koban et al., 2019; Nguyen et al., 2021). This particular coupled action-perception cycle is regarded as the touch feedback loop (Shamay-Tsoory and Eisenberger, 2021).

## The Role of Touch in Developing a Connection

At the centre of the TA is the harmonious relationship between the patient and the HCP in which the patient can trust, communicate, and have a mutual understanding of their goals and purpose, which patients with MSK disorders see as crucial to their treatment (Pinto et al., 2012; O’Keeffe et al., 2016; Miciak et al., 2019). Moreover, creating a collaborative relationship where the HCP and patient are seen as equals enables successful treatment outcomes, allowing HCPs to establish a meaningful connection (Miciak et al., 2018).

Therapeutic touch and hands-on techniques in MSK care help build an interpersonal connection by using the body as a pivot point (Miciak et al., 2018). An HCP’s role is to encourage the patient to reconnect to their body often through therapeutic touch to empathise how the body reacts and feels in response to the tactile stimulus from the HCP, essential to rehabilitation (Miciak et al., 2018; Geri et al., 2019). In patients with chronic LBP, the increased knowledge and awareness of their body will reaffirm the connection with the HCP as they feel their HCP understands their symptoms (Harman et al., 2011; Krueger, 2015, p. 263). This phenomenon encourages them to take ownership of their treatment plan (Miciak et al., 2018). Even the most rudimentary form of touch, such as, for example, a half-second of physical hand contact from a librarian, can increase positive perceptions of the library; similar acts have also helped establish the foundation for trust, compliance, cooperative and prosocial communication (Fisher et al., 1976; Morrison et al., 2010).

Touch is the most intuitive mode of expressing and detecting another’s emotional and mental states and sensory and communicative intentions, providing the context and clarity to ambiguous interactions (Hertenstein et al., 2006; McParlin et al., 2022). The different modes and manipulations of touch enable individuals to accurately infer at least six different emotions, including love, fear, and anger, with 48–83% accuracy (Herteinstein et al., 2006). As individuals become more precise at inferring another’s emotional and mental states, they also become better at establishing crucial elements of the TA such as common ground, trust and synchrony. Additionally, accurately inferring another’s hidden thoughts can simulate reward pathways as the individual minimises prediction errors, thereby increasing the enjoyment of the task (Beckes and Coan, 2011; Pan et al., 2017; Goldstein et al., 2018).

Health Care providers are in a unique position: it is often considered central and expected to clinically examine and therefore touch a wide area of the body, including the head and neck, generally reserved for more intimate or significant relationships (Geri et al., 2019). Additionally, it helps in the accuracy of HCP to infer the other individual’s emotions, detected through touch (Hertenstein et al., 2009; Geri et al., 2019).

Suvilehto et al. (2015) showed a correlation in the areas individuals will allow others to touch and the strength of the relationship. Therefore, HCPs who can touch more sensitive areas within their ethical boundaries are afforded a higher baseline level of trust and a greater opportunity to develop social bonds to build a stronger alliance and the overall analgesic effect. Therapeutic touch, when examining and validating their clinical condition, can frequently alleviate the patient’s anxiety and increase emotional support, empathy, mentalisation and be rewarding for all individuals (Gentsch et al., 2015). Additionally, it helps in emotional and communication regulation, as reflected in activating the right AIC, inferior parietal lobe and prefrontal cortex (Etkin et al., 2015; Goldstein et al., 2018; Redcay and Schilbach, 2019). It is the activation of the inferior parietal lobe that has been suggested to contribute to a significant role in the ability to infer another’s intentions and thoughts through observations, which may allow us to modulate and reduce the threat of noxious stimulus creating an analgesic effect (Kilner et al., 2007; Krahé et al., 2013; Von Mohr et al., 2018). Clinically, this has been shown in patients with acute postoperative pain, who reported decreased pain and anxiety after receiving massage therapy daily during their 5-day postoperative period (Mitchinson et al., 2007). Reduced anxiety associated with the dysfunction may aid in updating the autonomic and bottom-up interoceptive prediction errors that impair metacognitive motor homoeostatic and allostatic systems, which dictate dysfunction. This adds to the growing body of evidence that a more effective, stronger TA can improve clinical outcomes and patient satisfaction in treating MSK pain patients with manual therapy (Hush et al., 2011).

### Touch and Oxytocin

One of the most significant measures of bonding is the neurotransmitter oxytocin. It has been labelled as the social hormone as it enables the processing of social and non-social cues (Graustrella and MacLeod, 2012). Oxytocin also helps achieve biobehavioural synchrony and allostatic regulation and is released in therapeutic touch during hands-on techniques like massage (Uvnäs-Moberg, 2004). It has been argued that the somatosensory stimulations from touch-based therapies encourage anti-stress effects via the stimulating somatosensory pathways, subsequently activating the oxytocin system in the hypothalamus (Takahashi et al., 2021). Moreover, cervical spinal manipulations often used by HCPs in MSK care to treat acute non-specific mechanical neck pain have been shown to immediately affect neuromodulation, including oxytocin, potentially by modifying the neuropeptide expression (Lohman et al., 2019).

Moreover, multimodal sensory stimulation, including tactile touch, not only aid in the increase of cortical oxytocin levels but also helps with reduced social interaction, potentially increasing trust, and empathy essential for a successful TA (Green and Hollander, 2010; Yamasue et al., 2012; Zheng et al., 2014). Repeated stimulation of the CT afferents in the skin during affective touch increases the frequency and duration of the release of oxytocin receptors with tighter cross-links to dopamine and opioids; as well as regulating and increasing synchronisation within the autonomic nervous system (Löken et al., 2009; Ackerley et al., 2014; Pawling et al., 2017). Furthermore, a decrease in cortisol is observed when patients can recognise the repetition of similar techniques, exercises, appointment structure, and therapeutic touch with a familiar HCP (Uvnas-Moberg et al., 2015). Collectively, the evidence demonstrates that the repeated release of endogenous peptides, oxytocin, and opioids has therapeutic benefits, including analgesia, autonomic system regulation, and synchrony. Therefore, it can be argued that hands-on techniques still play a crucial role in MSK care.

Crucianelli et al. (2019) proposed that predictions of affective touch, could also be modulated by neuropeptides like oxytocin to optimise interoceptive attention. Therefore, oxytocin is likely to play a central role in interoception by modulating the precision of sensory stimuli in social contexts. The oxytocin-interoceptive nexus includes the multisensory effects of touch – somatosensory, thermoregulation and cardiorespiratory – on homoeostatic control (Quintana and Guastella, 2020; Kirsch et al., 2021). Oxytocin neuromodulation has been proposed as an aetiological factor in the failure to develop coherent models of “self” and “[m]other” (Quattrocki and Friston, 2014). Moreover, it has been suggested that the role of oxytocin is best characterised through allostasis; as it can help facilitate the dynamic adjustment and consolidation of homoeostatic setpoints, and is crucial to many of life’s essential survival needs (Quintana and Guastella, 2020). Therefore, the effective activation of the oxytocin system could help achieve positive physical and psychological clinical results through its ability to help patients adapt and encourage successful recovery (Takayanagi and Onaka, 2021). Additionally, oxytocin can help with analgesia due to its connection with decreased activity in the AIC in response to noxious stimuli, regulation of noxious threats, and increased activity in the prefrontal lobe (Kreuder et al., 2019). These findings are reflected in the use of therapeutic touch in MSK care, including in pregnant women with back and leg pain who experienced a reduction of pain and cortisol levels (Field, 2010; Mueller and Grunwald, 2021).

## Beyond the Health Care Provider-Patient Dyad: The Role of Hierarchy in Triadic Synchrony in the Clinical Encounter

To develop a robust and successful TA, HCPs need to work as a team to develop a collaborative relationship. In many circumstances, clinical interactions in MSK care are often triadic, with interactions occurring between the HCP, next of kin and the patient, i.e., an elderly relative or patient needing a translator. The active inference model of precision and weighting can be applied to clinical triadic situations through shared clinical goals, coordinated hands-on techniques, and therapeutic touch commonly used in MSK care. There is an increase in the coupling between the mirror neuron and mentalising systems in the brain during triadic social interactions, including the temporoparietal junction and prefrontal context, which are also influenced by therapeutic touch, including osteopathic manipulative treatment (Trapp et al., 2014; Tamburella et al., 2019). Crucially, the temporoparietal and medial temporal lobes are also intrinsic to the manipulation, validation, and reinforcement of adapting prior memories to specific contexts (Hasson et al., 2015).

The coordinated, complementary, triadic interactions increase the activation and coupling of mirror neurons and the inferior parietal lobe, which are engaged when two people make physical contact or participate in co-operative communication (Trapp et al., 2014; Miller et al., 2019). The three individuals will eventually synchronise their actions as they become more familiar with each other’s responses, increasing their communication intent. Therefore, triadic interactions activate both the mirror neuron and mentalisation systems, rather than the single activation of the mentalisation system observed in more direct interactions (Schilbach, 2010). The overlapping mapping of neural structures such as the pregenual anterior cingulate cortex, amygdala, and AIC, and the mentalisation systems are also activated similarly via tactile communication and hands-on care.

Touch is crucial to mentalisation, and the probabilistic inference frequently encountered through multisensory interoception, which is developed through the accumulation of multisensory autonomic and motor predictions of the body’s physiological states and coupling with the outside world (Fotopoulou and Tsakiris, 2017; Fotopoulou et al., 2022). The mirror neuron system suggests the utilisation of joint coding for one’s actions and perceptions with the understanding and ability to infer another individual’s actions, which is essential to co-operative communications (Koban et al., 2019). It is common for triadic situations to incorporate touch as a method of communication, combining the benefits of triadic interactions and touch into one scenario and increasing the overall synchrony. This can be applied to a clinical setting, when the HCP frequently determines the communication hierarchy delegating their expertise and advice to the next of kin, assisting with the patient’s regulation (O’Shea et al., 2019). While more verbal communications may be targetted at the next of kin than the patient, the patient receives significantly more stimulation from sensory information and repetition of movements throughout the treatment, utilising more direct tactile communication than the accompanying individual (Harman et al., 2011). We argue that understanding the mechanisms underpinning triadic communication and synchrony is crucial to providing effective person-centred MSK care, particularly in the care of the elderly, where the use of a translator is needed, and in contexts with a solid family-centred culture.

## Touch as a Method to Explore and Gain Expertise

According to patients, an essential quality of an HCP in MSK care is their expertise in the field (Peersman et al., 2013). Most patients believe their HCP has excellent clinical skills and is trustworthy, possibly due to their more precise prediction errors and priors on MSK disorders and associated symptoms such as pain, combined with their ability to help to resolve their symptoms (Cooper et al., 2008; Peiris et al., 2012; Del Baño-Aledo et al., 2014; Vasil et al., 2020). Despite a collaborative relationship, society’s hierarchical social strata reflect that as the HCP has superior knowledge, expertise, qualifications, and access to resources, they will always be seen higher in the hierarchical order of the relationship between HCP and patient (O’Shea et al., 2019). Thus, while strategies such as the person-centred approach allow for greater integration of patients’ voices in healthcare, HCPs will fundamentally make top-down decisions regarding their patients’ care.

Patients are frequently perceived to have an “interrogative motivation” in which they have a more receptive and motivated mindset to learn and create opportunities to adapt their current prior beliefs to some extent due to asymmetry entrainment (Harris and Corriveau, 2011; Harris et al., 2017). Patients with MSK-related pain typically receive cognitive reassurance through increased knowledge of their condition from their HCP – arguably, it helps develop more precise priors, decreases maladaptive beliefs, and improves patient confidence and condition management (Miciak et al., 2018; Cheung and Soundy, 2021). Touch and proprioception, i.e., haptics, is used to explore and gain information about the world around us. The haptic system enables us to discriminate and recognise objects through palpation (McLinden and McCall, 2002). Palpation is commonly used in MSK care. Despite its variable validity and reliability (see Nolet et al., 2021, for a recent review), patients believe that their HCPs can manually detect their clinical problem’s origin, thus explaining why they frequently expect an HCP to examine the area of dysfunction, particularly in subacute LBP.

## The Role of Touch in Overcoming Uncertainty by Creating a Safe Clinical Environment

Attachment theory suggests that an attachment figure can provide another individual with a strong sense of security Bowlby (1988). Through the lens of attachment theory, a therapist can take on this role and act as a “secure base” by instilling a sense of security in the clinical setting and thus strengthening the TA (Sauer et al., 2010). A safe environment and attachment can be created with the help of therapeutic touch and effective communication by acknowledging the clinical problem, receiving reassurance, the expectation of allostatic regulation and symptom modification, and encouraging the patient to engage in rehabilitation exercises, which they may not have felt confident to do (Bright et al., 2015; Taylor et al., 2015). Moreover, it is considered crucial and expected by patients with MSK disorders for a successful TA, rapport, installing patient confidence, motivation, ownership while showing empathy and reducing anxiety (Murray and Corney, 1991; Hill and Fritz, 2011; O’Keeffe et al., 2016; Cederbom et al., 2020; Cheung and Soundy, 2021). Therapeutic touch is also considered “comfort contact,” as it can contribute to patients feeling more comfortable with their HCP, especially in the case of LBP (GMC, 2020). Consequently, it can help to decrease stress through reassurance and achieving a secure attachment, a more vital relationship and a sense of safety that helps to regulate and accurately predict the physiological effects of stress and general health (Harlow and Zimmermann, 1959; Coan et al., 2006; Morrison, 2016b; López-Solà et al., 2019; Mühlenpfordt et al., 2020; Norholt, 2020). Compared to verbal reassurance, caring touch reduces stress significantly, reiterating its superiority in providing social support (Ditzen et al., 2007).

Individuals who experience persistent pain are more likely to suffer from anxiety, depression and fear-avoidance, thus a long-term obstacle to recovery that should be addressed (Linton and Shaw, 2011). Thus, increasing social support and a sense of safety can decrease the likelihood of the brain detecting and modulating potential threats, such as nociceptive signals, by increasing ventral medial prefrontal lobe activation, modulating pain and increasing the individual’s quality of life (Shamay-Tsoory and Eisenberger, 2021; Krahé et al., 2013). The evidence demonstrates that touch-based therapeutic interventions contribute to patients’ increased quality of life for many MSK conditions, including fibromyalgia, MSK, chronic pain, and headaches (Yuan et al., 2015; Crawford et al., 2016). Therefore, touch-based therapies are being recommended in the care of elderly patients with chronic MSK conditions, as increasing quality of life is often considered the main goal in symptomatic management, crucial to this demographic (Kopf, 2021). This speaks to the effect of touch of Aβ but also Aα, and CT afferents on the modulation of nociceptive signals at a subcortical level in conjunction with a high-level cortical response that can disrupt pain signalling, resulting in spinal gating that prevents signals from reaching the brain (Melzack, 1996; Mancini et al., 2015). Moreover, touch can moderate another individual’s level of pain, synchrony, and level of analgesia (Goldstein et al., 2017). The reduction in pain levels from hands-on care, particularly massage therapy, can be seen in a range of chronic pain conditions, including migraine headaches, fibromyalgia, LBP in different contexts, juvenile rheumatoid arthritis, and chronic paediatric conditions (Field et al., 2007; Suresh et al., 2008).

## The Role of Touch and Empathy Within the Clinical Encounter

Health Care provider and patients consider empathy as one of the fundamental elements required for establishing a TA, and significantly, it demonstrates positive effects on patient clinical outcomes and reducing distress by shared feelings and higher-order concerns with another, in the hope, they will help regulate them (Decety and Fotopoulou, 2015; O’Keeffe et al., 2016). Empathy is crucial in acknowledging the difficulties and sacrifices that the patient may face on the road to recovery by accepting and being willing to change or alter their prior and personal preferences to achieve full recovery (Bordin, 1979). Additionally, it contributes to the connection and synchrony between HCP and their patients by establishing a shared narrative, experience, emotional transfer, reinforcing the belief that everyone is the same. Moreover, it encourages the patient to relate to their current environment and discriminate their representations between self and others (Frijda and Mesquita, 1994).

When two people share similar emotions, they accurately infer the other’s actions, motivations, pain and suffering, thus reinforcing their relationship and priors by reducing the psychosocial barriers. Additionally, it helps patients with LBP and other MSK disorders to perceive their HCP as caring and empathic (Harman et al., 2011; Geri et al., 2019). In combination with repetitive dynamic hands-on techniques, the patient and HCP will often synchronise during spoken recall of a situation both had experienced – or when holding hands while experiencing noxious stimuli. This synchronisation is achieved through the activation of the Default Mode Network, high-level ventral temporal regions, intraparietal sulcus, pain-neuromatrix, anterior mid-cingulate cortex, AIC, inferior parietal lobe and IFG in both individuals, thus creating a prosocial effect (Preston, 2007; Romero et al., 2010; Goldstein et al., 2016; Chen et al., 2017; Korisky et al., 2020). Arguably, we are able to understand and share others’ emotions by partially processing them within our own emotional systems (Jackson et al., 2005; Vogt, 2005; Kilner et al., 2007; Friston, 2010; Lamm and Majdandžić, 2015).

Empathy is often associated with physiological responses in both the HCP and the patient, such as increased sympathetic nervous system activation, including skin conductance in chronic pain patients (Block, 1981). Moreover, more empathic individuals show more extradural synchrony and coupling, thus leveraging empathy – and learning the preferred coping strategies (Goldstein et al., 2017; Ellingsen et al., 2020; Reddan et al., 2020; Kozakevich Arbel et al., 2021; Shamay-Tsoory and Eisenberger, 2021). Indeed, it has been suggested that the degree of empathy demonstrated during touch correlates with the level of analgesia experienced by the partner through the toucher’s tactile stimulus (Goldstein et al., 2016; Korisky et al., 2020). All reaffirm the integral role that empathy plays in the TA and the patient management in MSK care.

## The Role of Repetition Within a Clinical Encounter in Aiding Synchrony

Clinical encounters are multifaceted and often have a repetitive structure, repeated each session to incorporate new external factors, changing symptom patterns and reactions or adjustments to the treatment (Pricop, 2016). Manual therapists generally have longer and more frequent appointments and patient continuity of care than a general medical practitioner (Miciak et al., 2018). In combination with repeated physical movements and hands-on care, these factors will influence the development of the TA. Moreover, they could contribute to the individual being more accurate, sensitive, and motivated to align with the HCP’s hidden mental states and recognise their unique therapeutic touch resulting in them potentially enhancing saliency, precision, synchrony, and confirming their priors more quickly (Tronick, 1989; Vasil et al., 2020). Additionally, it will increase the representation of social and cultural coherence and the likelihood of repeating the experience (Feldman, 2015; Begus et al., 2016). Finally, it bolsters the concept of repeated therapeutic touch, helping to promote self-awareness and synchrony – this could help explain why patients with high TA relationships adhere more closely to their physical rehabilitation programmes for many conditions, including neurological patients (Schönberger et al., 2006).

Repeated physical movements can putatively reinforce the patient’s pre-existing biological rhythms (Lester et al., 1985). It has been suggested that physiological synchrony, including HRV, is contingent on attention, similar processing of natural stimuli, and similar brain activity during memory processing (Pérez et al., 2021). As a result, the HCP and their patients’ physiological synchrony may be aided by the memory and expectations of previous hands-on care. Furthermore, it is imperative to MSK patients to have continuity of care and have “their HCP” who understands their body, activity levels and treatment preferences (Miciak et al., 2018).

The repetition of hands-on techniques during treatment increases sensitivity to pre-existing priors and allostatic regulation as a means of achieving homoeostasis. This is most notably seen bilaterally in the autonomic nervous system, precisely the cardiac and sympathetic tone. To this end, Tamburella et al. (2019) demonstrated that osteopathic manipulative therapy increased PCC perfusion significantly for 3 days, followed by an immediate decrease in resting cerebral perfusion within a cluster containing the Posterior Cingulate Cortex and Superior Parietal Lobe. The observed changes in cerebral perfusion suggest that touch may play a role in the observed improvements in sympathetic tone following sympathovagal modulation. Additionally, it has been hypothesised that behavioural synchrony and intimacy are associated with the evolution of the polyvagal system and its capacity to adapt to changes in our external environment (Porges, 2003, 2007). Several studies have demonstrated that the decreased heart rate and increased oxygen saturation following treatment could be sustained for 5–65 min after touch-based therapies (Lindgren et al., 2010; Manzotti et al., 2019). Touch has been shown to regulate physiological function and promote the development of precise embodied social behaviour and attachments, which can be influenced by the HCP’s treatment and personal predispositions (Hardin et al., 2020). The repetitive touch used in treatment can increase alpha EEG asymmetry, predominantly found in the left frontal hemisphere, and associated with emotional processing and cognitive maturation. Moreover, consciously processing audio-visual stimuli is associated with the degree and development of heart rate synchrony (Pérez et al., 2021). Therefore, we would argue that hands-on care supported by effective verbal and non-verbal communication strategies create an adequate multisensory environment to promote biobehavioural synchrony.

## Conclusion

In this article, we have proposed a model to explain the crucial role of touch and hands-on care in developing a robust TA, regulating allostasis and subsequently restoring homoeostasis in the field of MSK care. This model is based on AI and furnishes an integrative account of neurophysiological and biopsychosocial processes within a clinical encounter in MSK care. This formulation emphasises the foundational role of synchrony and cooperative communication between HCPs and their patients, hoping to engender successful clinical outcomes. While it is recognised that touch and skin contact is vital for survival – and has therapeutic benefits for patients – we consider a similar benefit for all individuals. This model reveals how touch and hands-on care can be used to revise an individual’s prior beliefs to create a person-centred care approach to promote allostasis and restore homoeostasis after an injury or to manage persistent pain and other functional medical symptoms. We have considered multiple feedback loops that offer potential mechanisms that can be leveraged during a treatment session. In short, the framework on offer enables HCPs to use touch and hands-on care techniques to strengthen the TA, minimise prediction errors (a.k.a., free energy), and thereby promote recovery from physical and psychological impairments.

Musculoskeletal care utilises therapeutic touch and communication to expose an individual to sensory stimuli and surprise, develop new associations and reactions to “overwrite” maladaptive priors and revise existing generative models (Stewart and Watt, 2008; Boettcher et al., 2016; Paulus et al., 2019; Smith et al., 2021). While this article focuses on touch-based interventions for treating and managing individuals suffering from MSK disorders, there are crossovers between mental health and MSK disorders – MSK related pain is associated with mood disorders (Bekhuis et al., 2015; Bohlen et al., 2021). Notwithstanding this association between chronic pain and mood disorders, MSK practitioners must acknowledge their limits of professional competence in mental health (Ianì, 2019; Bohlen et al., 2021). Psychotherapy also uses the body to elicit aspects related to embodied memory. Therefore, the complex, layered perceptions of memories and magnitude of uncertainty in understanding how bodily experience contributes to mental health must be respected (Ianì, 2019; Paoletti and Ben Soussan, 2019). HCPs must critically recognise the central and irreplaceable role of psychotherapy in treating and managing somatic and somatoform symptoms (Chemero, 2009; Gentsch and Kuehn, 2022).

Future research could consider the bidirectional neurobiological synchrony implied by the exchange of touch between HCP and patients in MSK care. This research line would help establish and consolidate the role of touch and hands-on care when characterising the complex and dynamic interactions during a clinical encounter. Moreover, an increased understanding of how touch and hands-on techniques could be implemented and manipulated to develop a successful TA could help dissolve the barriers encountered with patients who struggle to update their priors, particularly in multifactorial chronic MSK disorders.

## Data Availability Statement

The original contributions presented in this study are included in the article/supplementary material, further inquiries can be directed to the corresponding author/s.

## Author Contributions

ZM wrote the first draft of the manuscript. All authors contributed to the conception of the study and manuscript revision, and approved and accountable for the submitted manuscript.

## Conflict of Interest

GR leads education programmes on placebo, nocebo effects and contextual factors in healthcare to under- and postgraduate students along with private CPD courses. The remaining authors declare that the research was conducted in the absence of any commercial or financial relationships that could be construed as a potential conflict of interest.

## Publisher’s Note

All claims expressed in this article are solely those of the authors and do not necessarily represent those of their affiliated organizations, or those of the publisher, the editors and the reviewers. Any product that may be evaluated in this article, or claim that may be made by its manufacturer, is not guaranteed or endorsed by the publisher.
